# Septic arthritis and prosthetic joint infections: microbial spectrum and evolving resistance patterns

**DOI:** 10.5194/jbji-11-123-2026

**Published:** 2026-02-25

**Authors:** Merve Gürler, Füsun Kırca, Bedia Dinç

**Affiliations:** 1 Ankara Bilkent City Hospital, Department of Medical Microbiology, Ankara, Türkiye

## Abstract

**Background**: Septic arthritis (SA) and prosthetic joint infection (PJI) are severe musculoskeletal emergencies associated with rapid joint destruction, functional disability, and high mortality. Accurate microbiological diagnosis remains challenging, particularly in PJIs where biofilm formation reduces culture sensitivity. Local epidemiological data are essential to optimize empirical therapy and stewardship strategies. This study aimed to determine the distribution of microorganisms isolated from synovial fluid cultures and to evaluate antimicrobial resistance trends, with a direct comparison between native SA and PJI over a 5-year period. **Methods**: We retrospectively analyzed 3171 synovial fluid specimens collected between January 2020 and December 2024 at a tertiary referral hospital. Microorganisms were identified by MALDI-TOF MS, and antimicrobial susceptibility was tested with VITEK-2 according to EUCAST criteria. Resistance trends were assessed for major pathogens. **Results**: Overall, 651 samples (20.5 %) yielded growth, with significantly higher positivity in blood culture bottles than sterile containers (29.6 % vs. 16.1 %, p<0.001 ). PJIs accounted for 47.8 % of positive cultures. The most frequent pathogens were *Staphylococcus aureus* (33.6 %), coagulase-negative staphylococci (CoNS) (24.9 %), and *Pseudomonas aeruginosa* (8.6 %). CoNS ( p=0.017 ) and *E. faecalis* ( p=0.009 ) were significantly more common in PJIs. Methicillin resistance increased among *S. aureus* (20.0 % → 30.8 %) and remained high among CoNS (51 %–85 %). Extended-spectrum β -lactamase (ESBL) prevalence rose in *K. pneumoniae* (14.3 % → 42.1 %) and remained high in *E. coli* (57 %–80 %). All staphylococcal isolates remained susceptible to glycopeptides, linezolid, and daptomycin. **Conclusions**: Staphylococci remain the dominant pathogens in joint infections, with CoNS and *E. faecalis* strongly associated with PJIs. Rising methicillin resistance and ESBL-producing Enterobacterales highlight the need for careful empirical coverage, while preserved activity of last-line agents is reassuring. Routine inoculation into blood culture bottles significantly improves diagnostic yield.

## 1 Introduction

Septic arthritis (SA), also termed infectious arthritis, is a rheumatological emergency characterized by rapid joint destruction and considerable morbidity and mortality (Sharff et al., 2013). Bacterial invasion of the synovial space, most often through hematogenous spread, initiates acute inflammation that can swiftly damage cartilage and subchondral bone (Gigante et al., 2019; Shirtliff and Mader, 2002). SA can occur at any age, but it is most commonly seen in young children and older adults, with a consistent male predominance (Geirsson et al., 2008; Kaandorp et al., 1997a). The reported incidence ranges between 2 and 12 cases per 100 000 annually, with higher rates among individuals with comorbidities such as diabetes mellitus, chronic renal disease, malignancy, or immunosuppressive therapy (Momodu and Savaliya, 2022; Tande and Patel, 2014). Most patients present with acute monoarthritis, typically of the knee, and treatment delays beyond 24–48 h are strongly associated with irreversible joint damage, long-term disability, and increased mortality (Lieber et al., 2018).

Prosthetic joint infection (PJI) represents a particularly severe and complex form of SA, constituting one of the most devastating and costly complications of arthroplasty (Pina et al., 2019; Kurtz et al., 2008). Its incidence is projected to rise in parallel with an aging population, a higher prevalence of comorbid conditions, and the increasing number of joint replacement procedures performed worldwide (Ayoade et al., 2023). Management of PJI is especially difficult because of the ability of microorganisms to form biofilms on prosthetic surfaces. Biofilm-associated infections are protected from host immune responses and antimicrobial activity, often necessitating prolonged or repeated surgical interventions for eradication (Gbejuade et al., 2015).

Coagulase-negative staphylococci (CoNS) are among the leading pathogens implicated in PJI. While frequently regarded as contaminants in native joint infections, CoNS are now recognized as true pathogens in the prosthetic setting due to their biofilm-forming capacity and high rates of methicillin resistance (Tande and Patel, 2014). Contemporary studies confirm that *Staphylococcus aureus* (*S. aureus*) and CoNS are the predominant causative agents, although Gram-negative bacilli and enterococci are also encountered with clinically significant frequency (Suardi et al., 2024). Methicillin-resistant *S. aureus* (MRSA) has been associated with prolonged therapy, increased complications, and higher mortality (Kim et al., 2023). Similarly, the growing prevalence of methicillin-resistant *S. epidermidis* (MRSE) is of major concern, as these strains are often multi-drug resistant, leaving limited therapeutic options such as glycopeptides and linezolid (Bondarczuk et al., 2017).

Despite advances in diagnostic methods, the accurate identification of joint infections remains a challenge. The diagnosis of PJI, in particular, lacks universally accepted criteria and usually relies on a combination of clinical assessment, laboratory biomarkers, imaging, and microbiological findings. Synovial fluid aspiration and culture continue to be regarded as the diagnostic gold standard, as they enable pathogen identification and antimicrobial susceptibility testing (Parvizi et al., 2011). However, culture sensitivity is often reduced in patients with prior antibiotic exposure or in infections associated with biofilm formation, resulting in false-negative results (Trampuz and Zimmerli, 2006).

Given these challenges, timely and reliable microbiological characterization is crucial for guiding antimicrobial therapy and improving outcomes. Defining the microbial spectrum and resistance patterns in both native SA and PJI is essential not only for diagnostic accuracy but also for informing empirical treatment and stewardship practices. Yet, contemporary large-scale studies directly comparing native and prosthetic joint infections remain limited, particularly in regions with high antimicrobial resistance rates. The present study therefore aimed to investigate the distribution of microorganisms isolated from synovial fluid cultures and to evaluate their antimicrobial resistance profiles. By comparing native and prosthetic joint infections, we sought to highlight etiological differences, support optimization of empirical therapy, and contribute to improved clinical management of both conditions.

## 2 Methods

### 2.1 Study design and setting

This retrospective observational study was conducted at Ankara Bilkent City Hospital, a large tertiary referral center located in Ankara, the capital city of Türkiye. The study covered a 5-year period, from 1 January 2020 to 31 December 2024. Ankara Bilkent City Hospital has a capacity of 4050 beds and serves patients from both metropolitan and rural areas. With its specialized departments and advanced intensive care units (ICUs), the hospital plays a central role in the diagnosis and management of complex infectious diseases, including native and prosthetic joint infections (PJIs).

### 2.2 Inclusion and exclusion criteria

All synovial fluid samples submitted for microbiological culture during the study period were screened. Patients were excluded if demographic data were incomplete or if culture and susceptibility results were unavailable.

Case definitions were as follows:

**Prosthetic joint infection (PJI)**: PJI was defined according to the European Bone and Joint Infection Society (EBJIS) 2021 criteria (McNally et al., 2021). A case was defined as a PJI if it met at least one of the following criteria:

1. Presence of sinus tract communicating with the prosthesis,
2. Presence of purulence around the prosthesis,
3. A single positive culture yielding a pathogen microorganism (e.g., *S. aureus*),
4. Elevated synovial fluid white blood cell count ( >3000 cells per µ L) or positive leukocyte esterase strip test ( ++ or +++ ) in the absence of a underlying inflammatory arthropathy,
5. Positive histopathological findings consistent with infection in periprosthetic tissue.

**Native septic arthritis (SA)**: SA was defined by the presence of acute clinical signs of arthritis (pain, swelling, erythema, warmth) in a native joint, combined with either:

1. A positive synovial fluid culture, or
2. A synovial fluid white blood cell count >50000 cells per µ L in the absence of an alternative diagnosis.

**Definition of contaminants**: Culture-positive results were defined as contaminants if Gram staining yielded no microorganisms, the synovial fluid leukocyte count was <2000 per mm^3^, and there were no clinical signs of arthritis (Ottink et al., 2019).

For coagulase-negative staphylococci (CoNS), isolates were considered clinically significant only in the presence of prosthetic joints, a documented history of joint surgery, or clear clinical evidence of infection at the involved joint. Single positive CoNS cultures without supportive clinical findings were regarded as contaminants and excluded from analysis.

For patients with multiple culture submissions, only the first positive culture per patient was analyzed.

### 2.3 Patient population

A total of 3171 unique patients were included, each contributing one synovial fluid specimen. Demographic and clinical variables, including age, sex, joint type (native vs. prosthetic), and referring clinical department, were retrieved from the hospital information system. Patients were stratified into pediatric ( ≤18 years) and adult ( >18 years) subgroups. For PJI cases, the anatomical site of the prosthesis (knee, hip, or shoulder) was additionally recorded.

### 2.4 Sample collection and processing

Synovial fluid specimens were obtained by aseptic aspiration. Depending on routine clinical practice, samples were either transported in sterile containers or directly inoculated into aerobic blood culture bottles at the bedside. Blood culture bottles were incubated in the BacT/ALERT^®^ 3D system (bioMérieux, France) for up to 5 d.

Upon positivity, and for all samples received in sterile containers, samples were subcultured onto 5 % sheep blood agar, chocolate agar, and Eosin methylene blue (EMB) agar, and incubated at 37 °C in 5 % CO_2_ for 48–72 h. Bacterial isolates were identified using the VITEK^®^ MS system (bioMérieux, France), based on matrix-assisted laser desorption/ionization time-of-flight mass spectrometry (MALDI-TOF MS).

### 2.5 Antimicrobial susceptibility testing

Susceptibility testing was performed with the automated VITEK^®^ 2 system (bioMérieux, France), which determines minimum inhibitory concentrations (MICs) and provides interpretative categories. Multi-drug-resistant phenotypes were defined as follows:

- Extended-spectrum β -lactamase (ESBL) production in Enterobacterales,
- Methicillin resistance in *Staphylococcus aureus* (MRSA),
- Methicillin resistance in CoNS (MR-CoNS).

Colistin susceptibility was determined by broth microdilution in accordance with ISO 20776-1 standards. Results were interpreted according to the annually updated breakpoints of the European Committee on Antimicrobial Susceptibility Testing (EUCAST).

### 2.6 Data collection

Culture positivity rates were calculated overall and stratified by clinical department and transport method (sterile container vs. blood culture bottle). The distribution of pathogens was compared across adult and pediatric patients, and between native and prosthetic joints. Annual antimicrobial resistance trends were analyzed for the six most frequently isolated organisms: *S. aureus*, CoNS, *Enterococcus faecalis* (*E. faecalis*), *Pseudomonas aeruginosa* (*P. aeruginosa*), *Klebsiella pneumoniae* (*K. pneumoniae*), and *Escherichia coli* (*E. coli*).

### 2.7 Statistical analysis

All analyses were performed using SPSS Statistics version 22.0 (IBM Corp., Armonk, NY, USA). Descriptive statistics were applied to summarize categorical and continuous variables. Continuous variables were expressed as medians with ranges, while categorical data were compared using Pearson's $\chi^{2}$ or Fisher's exact test, as appropriate. A two-sided p value < 0.05 was considered statistically significant. Annual antimicrobial resistance rates were summarized descriptively by study year for the most frequently isolated pathogens. No formal statistical trend analysis was performed, as the primary objective was to describe year-to-year changes in resistance patterns rather than to test for temporal trends.

### 2.8 Ethical approval

This study was approved by the Ankara Bilkent City Hospital No. 1 Medical Research Scientific and Ethical Evaluation Board (TABED) (approval number TABED 1/1625/2025, dated 27 August 2025). The study was conducted in accordance with the principles of the Declaration of Helsinki and applicable national research ethics regulations. Given the retrospective design and the use of anonymized routine laboratory data, the requirement for written informed consent was formally waived by the ethics committee.

**Figure 1:**
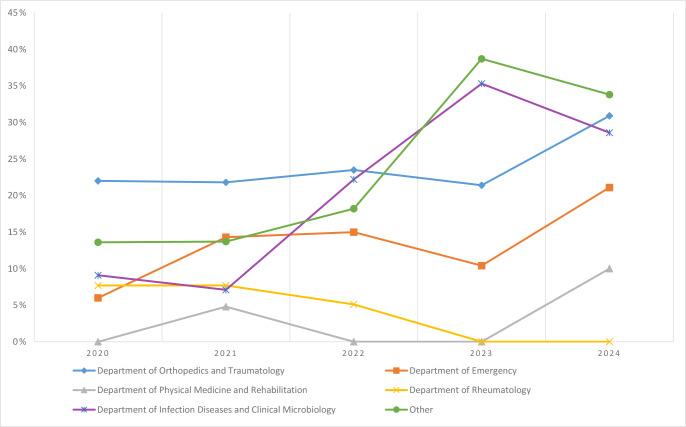
Annual resistance rates of culture positivity rates across clinical departments (2020–2024).

## 3 Results

### 3.1 Study population and samples

Between 1 January 2020 and 31 December 2024, a total of 3171 synovial fluid samples were processed, originating from 1654 females (52.1 %) and 1517 males (47.9 %). Adults contributed 2949 samples (93.0 %) and children 222 samples(7.0 %) (Table 1). Most requests were from the Department of Orthopedics and Traumatology ( n=1937 ; 61.1 %), followed by the departments of Emergency ( n=558 ; 17.6 %), Physical Medicine and Rehabilitation ( n=143 ; 4.5 %), Rheumatology ( n=139 ; 4.4 %), and Infectious Diseases and Clinical Microbiology ( n=74 ; 2.3 %). Other departments collectively submitted 320 samples (10.1 %). Transport methods included sterile containers ( n=2127 ; 67.1 %) and blood culture bottles ( n=1044 ; 32.9 %).

**Table 1:** Number of synovial fluid culture samples and demographic characteristics of patients by year.

Year			2020	2021	2022	2023	2024	Total
								n (%)
Total number of synovial fluid samples			422	582	761	735	671	3171 (100 %)
Patients	Gender	Female	204	258	412	428	352	1654 (52.2 %)
		Male	218	324	349	307	319	1517 (47.8 %)
	Age	Children	32	45	50	55	40	222 (7 %)
	groups	Adults	390	537	711	680	631	2949 (93 %)
Number of positive synovial fluid samples			70	103	146	146	186	651 (100 %)
Patients	Gender	Female	31	41	80	78	98	328 (50.4 %)
		Male	39	62	66	68	88	323 (49.6 %)
	Age	Children	2	3	11	17	12	45 (6.9 %)
	groups	Adults	68	100	135	129	174	606 (93.1 %)

Children: 1–18 years, adults: >18 years.

### 3.2 Culture positivity and prosthetic joint infection characteristics

Overall, 651 of 3171 samples (20.5 %) yielded growth. Among positives, 328 (50.4 %) were from females and 606 (93.1 %) were from adults (Table 1). Culture positivity varied considerably across departments. The highest yields were observed in Orthopedics and Traumatology(468/1937; 24.2 %), and Infectious Diseases and Clinical Microbiology (16/74; 21.6 %), whereas positivity was much lower in Rheumatology (5/139; 3.6 %), and Physical Medicine and Rehabilitation (3/143; 2.1 %). The Department of Emergency yielded 78 positive cultures (14.0 %), and other departments together accounted for 81 (25.3 %) (Table 2, Fig. 1).

**Table 2:** Descriptive analysis for synovial fluid culture-positive samples based on years 2020–2024.

			2020	2021	2022	2023	2024	Total
			( n=70 ,	( n=103 ,	( n=146 ,	( n=146 ,	( n=186 ,	( n=651 ,
			16.6 %)	17.7 %)	19.2 %)	19.7 %)	27.7 %)	20.5 %)
			n (%)	n (%)	n (%)	n (%)	n (%)	n (%)
Sample	Sterile container		38	51	76	83	94	342
transport	( n=2127 )		(11.6 %)	(12.1 %)	(14.7 %)	(18 %)	(23.3 %)	(16.1 %)
method	Blood culture bottles		32	52	70	63	92	309
	( n=1044 )		(33.3 %)	(32.3 %)	(28.6 %)	(22.9 %)	(34.4 %)	(29.6 %)
Clinics	Department of Orthopedics and Traumatology		58	72	107	97	134	468
	( n=1937 )		(22 %)	(21.8 %)	(23.5 %)	(21.4 %)	(30.9 %)	(24.2 %)
	Department of Emergency		4	20	19	12	23	78
	( n=558)		(6 %)	(14.3 %)	(15 %)	(10.4 %)	(21.1 %)	(14 %)
	Department of Physical Medicine and Rehabilitation		0	1	0	0	2	3
	( n=143 )		(0 %)	(4.8 %)	(0 %)	(0 %)	(10 %)	(2.1 %)
	Department of Rheumatology		1	2	2	0	0	5
	( n=139 )		(7.7 %)	(7.7 %)	(5.1 %)	(0 %)	(0 %)	(3.6 %)
	Department of Infection Diseases and Clinical Microbiology		1	1	4	6	4	16
	( n=74)		(9.1 %)	(7.1 %)	(22.2 %)	(35.3 %)	(28.6 %)	(21.6 %)
	Others		6	7	14	31	23	81
	(n= 20)3		(13.6 %)	(13.7 %)	(18.2 %)	(38.7 %)	(33.8 %)	(25.3 %)
Organism	Gram-	*S. aureus*	25	36	53	53	52	219
	positive		(35.7 %)	(34.9 %)	(36.3 %)	(36.3 %)	(27.9 %)	(33.6 %)
		CoNS	12	33	38	39	40	162
			(17.1 %)	(32 %)	(26 %)	(26.7 %)	(21.5 %)	(24.9 %)
		*E. faecalis*	0	1	10	10	11	32
			(0 %)	(1 %)	(6.8 %)	(6.8 %)	(5.9 %)	(4.9 %)
	Gram-	*P. aeruginosa*	4	3	9	19	21	56
	negative		(5.7 %)	(2.9 %)	(6.2 %)	(13 %)	(11.3 %)	(8.6 %)
		*K. pneumoniae*	7	5	4	13	19	48
			(10 %)	(4.8 %)	(2.7 %)	(8.9 %)	(10.2 %)	(7.4 %)
		*E. coli*	5	3	13	13	7	43
			(7.1 %)	(2.9 %)	(8.9 %)	(8.9 %)	(3.8 %)	(6.6 %)
	Others		18	25	19	9	50	121
			(25.7 %)	(24.3 %)	(13 %)	(6.2 %)	(26.9 %)	(18.6 %)

*S. aureus*: *Staphylococcus aureus*, CoNS: coagulase-negative staphylococci, *E. faecalis*: *Enterococcus faecalis*, *P. aeruginosa*: *Pseudomonas aeruginosa*, *K. pneumoniae*: *Klebsiella pneumoniae*, *E. coli*: *Escherichia coli*.

The positivity rate was significantly higher for blood culture bottles than sterile containers: 29.6 % (309/1044) vs. 16.1 % (342/2127) ( p<0.001 ) (Table 2).

Of all culture-positive cases, nearly half (311/651; 47.8 %) were classified as PJIs, highlighting their major contribution to the burden of joint infections in this tertiary center. Among PJI cases, 68.2 % occurred in females, with a mean age of 67.4 years (range 10–99). The distribution of affected prostheses was knee (57.9 %; n=180 ), hip (38.9 %; n=121 ), and shoulder (3.2 %; n=10 ) (Table 3).

**Table 3:** Demographic and clinical characteristics of patients with culture-positive prosthetic joint infections.

Sex,	Female	212 (68.2 %)
n (%)	Male	99 (31.8 %)
Age (min–max)		67.4 (10–99)
Prosthetic joint site,	Knee	180 (57.9 %)
n (%)	Hip	121 (38.9 %)
	Shoulder	10 (3.2 %)

### 3.3 Distribution of microorganisms

The six most frequently isolated pathogens were *S. aureus* (33.6 %), CoNS (24.9 %), *P. aeruginosa* (8.6 %), *K. pneumoniae* (7.4 %), *E. coli* (6.6 %), and *E. faecalis* (4.9 %). Collectively, these organisms accounted for approximately 86 % of all isolates (Table 2).

**Figure 2:**
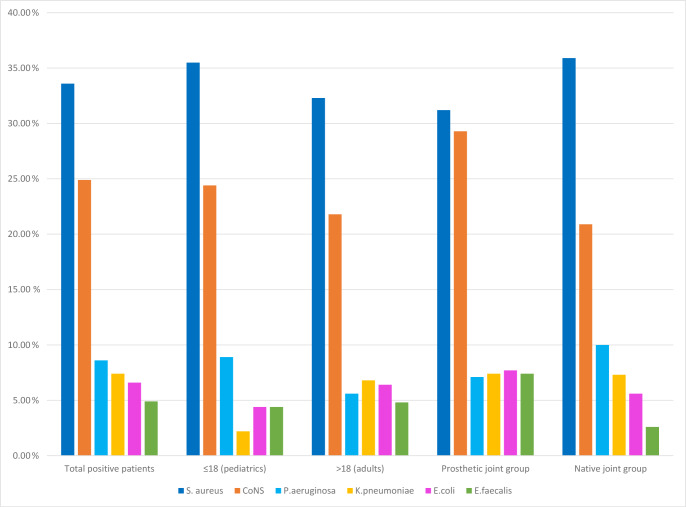
Distribution of major causative microorganisms according to age group and joint type.

Comparative analysis revealed no statistically significant difference between adult and pediatric groups in terms of isolated microorganisms. Crucially, when stratified by infection type, the microbiological etiology revealed significant disparities. CoNS ( p=0.017 ) and *E. faecalis* ( p=0.009 ) were significantly more frequent in PJIs compared with native joint infections. No significant difference was detected for *S. aureus*, *P. aeruginosa*, *E. coli*, or *K. pneumonia* (Fig. 2).

**Figure 3:**
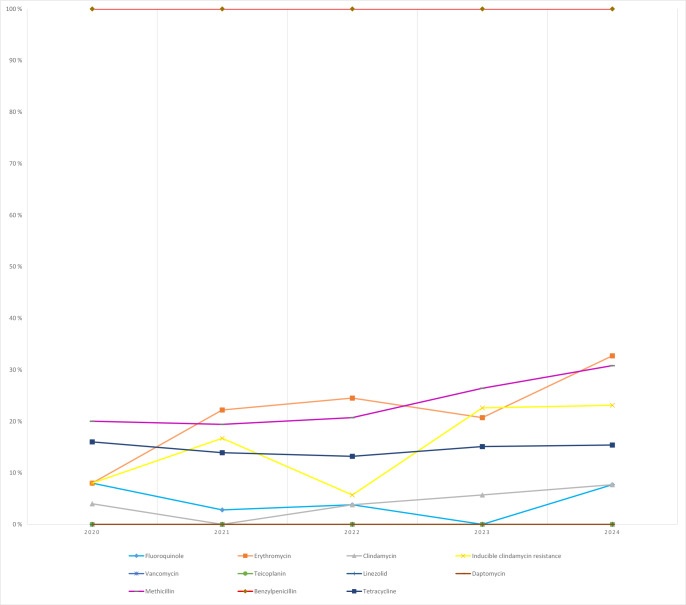
Temporal distribution of antibiotic resistance rates in *Staphylococcus aureus* isolates.

### 3.4 Annual antimicrobial resistance rates (2020–2024)

**Figure 4:**
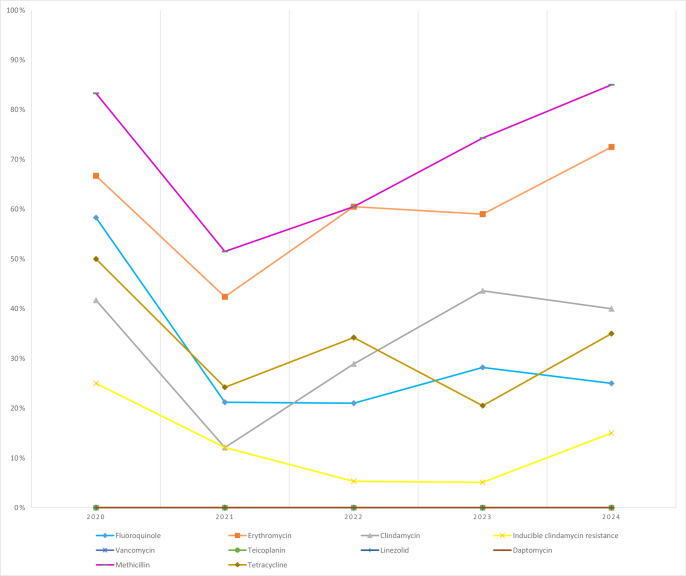
Temporal distribution of antibiotic resistance rates in CoNS isolates.

**Figure 5:**
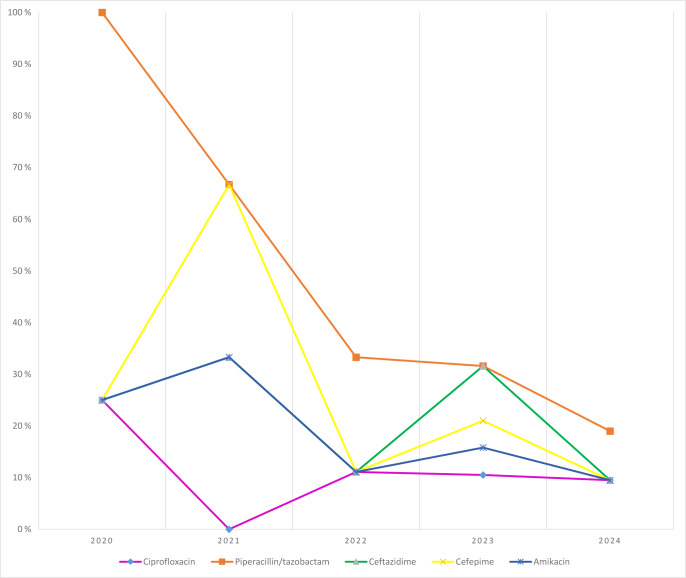
Temporal distribution of antibiotic resistance rates in *Pseudomonas aeruginosa* isolates.

**Figure 6:**
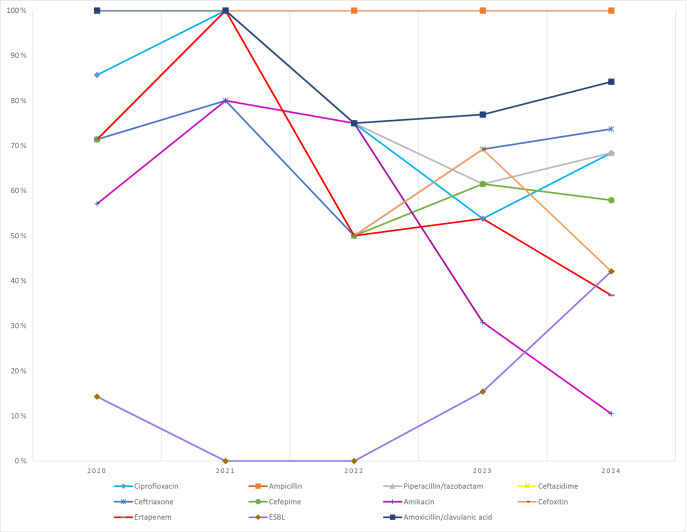
Temporal distribution of antibiotic resistance rates in *Klebsiella pneumoniae* isolates.

**Figure 7:**
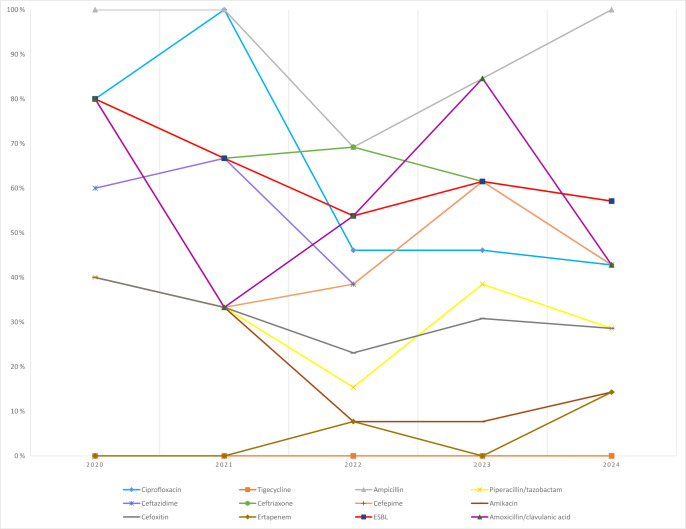
Temporal distribution of antibiotic resistance rates in *Escherichia coli* isolates.

- *S. aureus*: methicillin resistance increased from 20.0 % (2020) to 30.8 % (2024) over the study period. Erythromycin resistance increased steadily, reaching 32.7 % in 2024; inducible clindamycin resistance also increased to 23.1 %. No resistance was detected to vancomycin, teicoplanin, linezolid, or daptomycin in any year (Fig. 3).
- CoNS: methicillin resistance remained high ( ≈51 %–85 %) throughout. Erythromycin resistance reached 72.5 % (2024). All isolates were susceptible to glycopeptides and linezolid across years (Fig. 4).
- *E. faecalis*: all isolates were susceptible to tested agents during the study period.
- *P. aeruginosa*: piperacillin/tazobactam resistance decreased from 100 % (2020) to 19 % (2024). Resistance to ciprofloxacin, ceftazidime, cefepime, and amikacin fluctuated typically by 10 %–30 % annually (Fig. 5).
- *K. pneumoniae*: resistance remained high across most β -lactams. ESBL production increased from 14.3 % (2020) to 42.1 % (2024). Carbapenem resistance rose over time, reaching 36.8 % for ertapenem (Fig. 6).
- *E. coli*: ESBL rates ranged from 57 % to 80 %. Resistance to third-generation cephalosporins (e.g., ceftriaxone/ceftazidime) exceeded 50 % in most years, whereas carbapenem resistance remained limited ( ≤14.3 %) (Fig. 7).

## 4 Discussion

SA and PJI are medical emergencies due to their potential for rapid cartilage destruction and long-term disability. Although diagnosis relies on clinical, laboratory, and imaging findings, synovial fluid culture remains the cornerstone despite limited sensitivity and delayed turnaround (Parvizi et al., 2011). In PJI, biofilm formation complicates management and increases the risk of treatment failure (Zimmerli et al., 2004; Gbejuade et al., 2015). Evidence from randomized trials on optimal antibiotic regimens in SA is limited, underscoring the need for strategies informed by local microbiology and resistance trends (Gjika et al., 2019). In this context, our 5-year study analyzed synovial fluid cultures, compared native and prosthetic infections, and described year-to-year changes in antimicrobial resistance patterns.

Overall, 20.5 % of synovial fluid samples yielded microbial growth, lower than positivity rates commonly reported in the literature (42 %–56 %) (Geirsson et al., 2008; Kaandorp et al., 1997a; Ravn et al., 2023; Talsma et al., 2021). This discrepancy likely reflects prior antibiotic exposure, sampling variability, and the absence of adjunctive techniques such as sonication. The low yield emphasizes the continued relevance of culture surveillance and the potential value of complementary molecular diagnostics. Adults accounted for most culture-positive cases (93.1 %), and pediatric cases were comparatively infrequent, consistent with the predominance of PJIs in older populations and the increasing burden of SA with age (Geirsson et al., 2008; Kaandorp et al., 1997b). This distribution likely reflects the inclusion of PJIs, a condition seen primarily in adults. Unlike population-based reports of male predominance in native SA (Geirsson et al., 2008; Mathews et al., 2010), we did not observe a marked sex difference, which may be explained by the inclusion of both native and prosthetic infections, and the demographic profile of arthroplasty recipients (Lenguerrand et al., 2018; Ren et al., 2021; De Mauro et al., 2024).

Positivity rates varied across departments. The highest yields were observed in the Department of Orthopedics and Traumatology (24.2 %) and the Department of Infectious Diseases and Clinical Microbiology (21.6 %), reflecting targeted sampling and frequent intraoperative cultures (Shamdasani et al., 2023). In contrast, lower yields in the Department of Emergency (14.0 %) likely resulted from heterogeneous case presentations and prior empiric therapy (Puzzitiello et al., 2024), whereas the very low rates in Rheumatology (3.6 %), and Physical Medicine and Rehabilitation (2.1 %) mainly reflected cultures obtained to exclude infection in predominantly non-infectious joint diseases (Pal et al., 1997).

A key practical finding was the improved recovery rate when synovial fluid was inoculated into blood culture bottles compared with sterile containers. This observation aligns with prior reports suggesting that higher inoculum volumes and antibiotic-neutralizing media can enhance detection (Cohen et al., 2020; Font-Vizcarra et al., 2010; Li et al., 2019). Together, these findings support routine inoculation of synovial fluid into blood culture bottles as a simple and implementable measure to increase diagnostic sensitivity and potentially reduce false-negative cultures in routine practice.

Nearly half of culture-positive cases were PJIs, characterized by higher age, female predominance, and knee localization, consistent with prior registry data (Nair et al., 2017; Tai et al., 2022; Khakzad et al., 2022). The microbial spectrum was dominated by staphylococci. *S. aureus* remained the most frequent pathogen overall, confirming its central role across native SA and PJI (Tande and Patel, 2014; Arieli et al., 2021). CoNS were especially frequent in PJIs, underscoring their pathogenic significance in the prosthetic setting through biofilm formation and device-related persistence (Arieli et al., 2021; Chan et al., 2020). Gram-negative bacilli accounted for 22.6 % of isolates, led by *P. aeruginosa*, *K. pneumoniae*, and *E. coli*, again consistent with published series (Tsaras et al., 2012). *E. faecalis* was isolated in 4.9 % of cases, higher than the 1.9 % reported by Hasbek and Çubuk (2022), and, together with CoNS, was significantly more common in PJI, confirming its strong association with prosthetic material (Renz et al., 2019; Linke et al., 2022). In contrast, *S. aureus* and Gram-negative bacilli were more evenly distributed between native and prosthetic infections, consistent with prior reports (Linke et al., 2022; Talsma et al., 2021; Gbejuade et al., 2015). Pathogen profiles did not vary by age, consistent with Alexandersson et al. (2024), suggesting that clinical differences across age strata are more likely driven by host factors and comorbidities than by major shifts in microbial etiology.

Year-to-year resistance patterns revealed clinically relevant changes. Methicillin resistance among *S. aureus* increased over the study period, consistent with broader reports and reinforcing the need to ensure appropriate empirical coverage, particularly given the association between methicillin resistance and adverse outcomes in SA (Ben-Chetrit et al., 2020; Dubost et al., 2014; Kim et al., 2023). Increasing macrolide resistance and inducible clindamycin resistance were also observed, paralleling international findings (Wu et al., 2023). In contrast, glycopeptides, linezolid, and daptomycin maintained full activity, in agreement with European reports (Ritchie et al., 2010; Markwart et al., 2021), supporting their continued role as cornerstone agents for severe Gram-positive joint infections.

Methicillin resistance among CoNS remained consistently high, in line with Turkish and international reports (Hasbek and Çubuk, 2022; Hu et al., 2021). Together with their biofilm capacity, this poses a major therapeutic challenge (Gbejuade et al., 2015). Erythromycin resistance was also high, reaching 72.5 % in 2024, which is consistent with patterns reported elsewhere (Hasbek and Çubuk, 2022; Hu et al., 2021). In contrast, retained susceptibility to glycopeptides and linezolid mirrors previous reports (Hellmark et al., 2009; Suardi et al., 2024) and emphasizes the importance of preserving these last-line options through stewardship. *E. faecalis* isolates remained universally susceptible in our dataset (Martin et al., 2023); nevertheless, their increased frequency in PJIs warrants clinical vigilance, particularly in mixed infections and in settings with prolonged antimicrobial exposure (Renz et al., 2019).

Resistance patterns among Gram-negative organisms were heterogeneous. For *P. aeruginosa*, piperacillin–tazobactam resistance decreased across the study years; however, this pattern should be interpreted cautiously, as changes in EUCAST breakpoints may influence apparent year-to-year resistance rates independent of true epidemiological shifts (Ourghanlian et al., 2022; Johnson et al., 2022). Similar breakpoint-related variability has been described for ceftazidime, cefepime, and ciprofloxacin (Wolfensberger et al., 2013). Amikacin resistance remained relatively low, consistent with observations from other regions (Abdeta et al., 2023). These findings highlight the need to interpret longitudinal resistance data in the context of evolving interpretive standards and local stewardship practices.

*K. pneumoniae* demonstrated substantial β -lactam resistance, and ESBL production increased over the study period (Ramatla et al., 2023). Carbapenem resistance remained clinically important, with variability across years that likely reflect patient factors, antimicrobial use, infection control, and evolving EUCAST breakpoints. These findings parallel European Centre for Disease Prevention and Control (ECDC) surveillance data showing high carbapenem resistance in parts of Europe data (ECDC, 2025).

*E. coli* also showed high ESBL rates and marked third-generation cephalosporin resistance, whereas carbapenem resistance remained relatively limited, similar to data reported from Pakistan (Ali Khan et al., 2025) but differing from a Romanian PJI cohort reporting the universal susceptibility of *E. coli* (Dragosloveanu et al., 2025). The high ESBL prevalence in our series most likely reflects regional prescribing practices and ecological pressures.

This study has limitations. The retrospective design may have resulted in incomplete clinical data, including unmeasured prior antibiotic exposure, which could influence culture yield and pathogen recovery. The reliance on conventional culture without adjunctive methods such as sonication or molecular assays may have underestimated the contribution of some organisms. As a single-center study, generalizability to other settings is limited. In addition, year-to-year comparisons of resistance rates may be influenced by changes in EUCAST breakpoints over time. These limitations support the need for prospective multi-center studies incorporating standardized sampling, enhanced diagnostics, and detailed clinical metadata.

In conclusion, our 5-year analysis confirms the predominance of staphylococci in joint infections and highlights the strong association of CoNS and *E. faecalis* with PJIs. Increasing methicillin resistance among staphylococci and the high prevalence of ESBL-producing Enterobacterales emphasize the importance of careful empirical coverage tailored to local epidemiology; whereas preserved activity of glycopeptides, linezolid, and daptomycin is reassuring for severe Gram-positive infections. Finally, routine inoculation of synovial fluid into blood culture bottles substantially improves diagnostic yield and represents a practical evidence-based strategy to strengthen routine diagnostic workflows and optimize patient management.

## Data Availability

The datasets generated and analyzed during the current study are not publicly available due to patient confidentiality and institutional data protection regulations but are available from the corresponding author on reasonable request, subject to ethical approval.
